# Recent Progress in the Energy Harvesting Technology—From Self-Powered Sensors to Self-Sustained IoT, and New Applications

**DOI:** 10.3390/nano11112975

**Published:** 2021-11-05

**Authors:** Long Liu, Xinge Guo, Weixin Liu, Chengkuo Lee

**Affiliations:** 1 Department of Electrical and Computer Engineering, National University of Singapore, 4 Engineering Drive 3, Singapore 117576, Singapore; eleliulo@nus.edu.sg (L.L.); guoxg@u.nus.edu (X.G.); elelwei@nus.edu.sg (W.L.); 2 Center for Intelligent Sensors and MEMS, National University of Singapore, Block E6 #05-11, 5 Engineering Drive 1, Singapore 117608, Singapore; 3 NUS Suzhou Research Institute (NUSRI), Suzhou Industrial Park, Suzhou 215123, China; 4 NUS Graduate School—Integrative Sciences and Engineering Program (ISEP), National University of Singapore, Singapore 119077, Singapore

**Keywords:** energy harvester (EH), self-powered sensor, internet of things (IoT), triboelectric nanogenerator (TENG), piezoelectric nanogenerator (PENG)

## Abstract

With the fast development of energy harvesting technology, micro-nano or scale-up energy harvesters have been proposed to allow sensors or internet of things (IoT) applications with self-powered or self-sustained capabilities. Facilitation within smart homes, manipulators in industries and monitoring systems in natural settings are all moving toward intellectually adaptable and energy-saving advances by converting distributed energies across diverse situations. The updated developments of major applications powered by improved energy harvesters are highlighted in this review. To begin, we study the evolution of energy harvesting technologies from fundamentals to various materials. Secondly, self-powered sensors and self-sustained IoT applications are discussed regarding current strategies for energy harvesting and sensing. Third, subdivided classifications investigate typical and new applications for smart homes, gas sensing, human monitoring, robotics, transportation, blue energy, aircraft, and aerospace. Lastly, the prospects of smart cities in the 5G era are discussed and summarized, along with research and application directions that have emerged.

## 1. Introduction

In the past few decades, the electronic devices distributed in our daily life have received enormous change along with the evolution of sensing technology. The microelectromechanical systems (MEMS) technology has promoted billions of miniature sensors with various functions, compact size, and low power consumption [1,2], through which the current electronic devices are able to become lighter, smarter, and more helpful. Simultaneously, with the flourishing of wireless communication technology, the billions-level massive number of sensors can be linked with each other in an unprecedented way in the coming 5 G era [3,4,5]. We can foresee that the internet of things (IoT), Industry 4.0, the smart city, smart home, etc., will step into and greatly improve our lives in the near future, making our lives more convenient and intelligent [6,7,8,9,10]. However, such development also brings a great challenge regarding the power supply for that enormous wireless sensor nodes, while some of them are even placed in harsh environments such as inside the human body, on the high buildings and bridges, and in mines and tunnels [11,12,13,14,15]. Traditionally, batteries are the primary practical choices to power electronic devices, while their drawbacks become more obvious when integrated with miniature sensors that are applied in multiple environments, such as the difficulty of regular replacing and recharging caused by limited power density, biological incompatibility to the human body, and high contamination to the environment [16,17,18]. These drawbacks greatly hinder the further development of wireless sensor nodes and IoT systems and, therefore, correspondingly raise new research directions toward self-sustainability, which is being achieved through energy harvesting and self-powered sensing technology [19,20,21,22].

**Figure 1:**
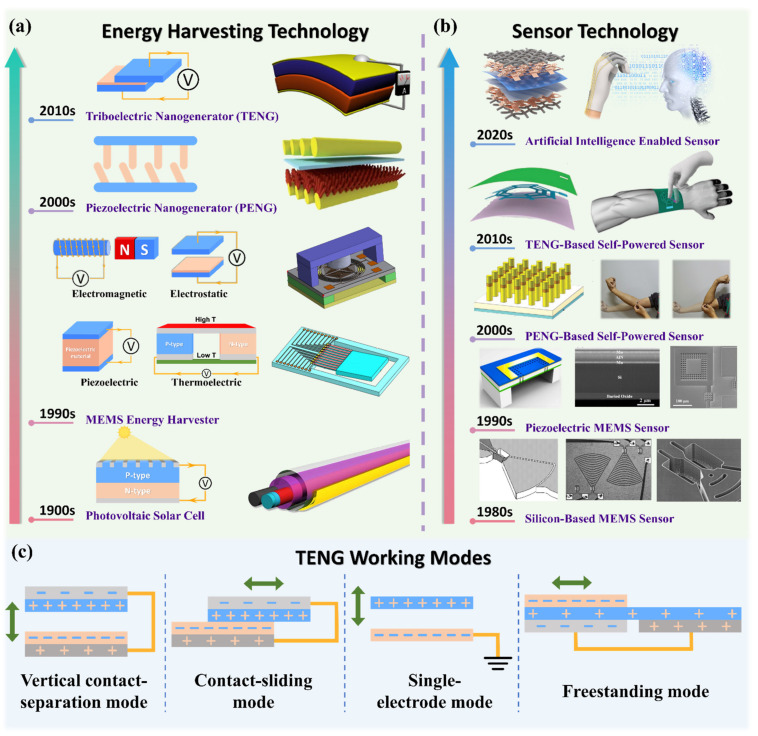
(**a**) Roadmap for the energy harvesting technology and typical works for illustration: 1900s—photovoltaic solar cell and a textile fiber-shaped dye-sensitized solar cell for wearable electronics (Reprinted with permission from ref. [27]. Copyright 2016 American Association for the Advancement of Science); 1990s—MEMS energy harvesters based on electromagnetic, electrostatic, piezoelectric, and thermoelectric and an electromagnetic MEMS VEH (Reprinted with permission from ref. [24]. Copyright 2012 IOP Publishing, and a piezoelectric MEMS VEH) (Reprinted with permission from ref. [26]. Copyright 2011 IEEE); 2000s—piezoelectric nanogenerator based on piezoelectric nanowires and a ZnO nanowires-based PENG on a textile platform (Reprinted with permission from ref. [23]. Copyright 2012 Royal Society of Chemistry); 2010s—triboelectric nanogenerator and the firstly proposed flexible TENG device (Reprinted with permission from ref. [25]. Copyright 2012 Elsevier). (**b**) Roadmap for the sensor technology and typical works for illustration: 1980s—silicon-based MEMS sensor and a MEMS piezoresistive accelerometer (Reprinted with permission from ref. [62]. Copyright 2000 IEEE); 1990s—piezoelectric-based MEMS sensor and a MEMS piezoelectric micromachined ultrasonic transducer (Reprinted with permission from ref. [63]. Copyright 2015 IEEE); 2000s—PENG-based self-powered sensor and a GaN microwire array-based wearable PENG strain sensor (Reprinted with permission from ref. [65]. Copyright 2020 John Wiley & Sons); 2010s—TENG-based self-powered sensor and a spider-net-coding interface with single-electrode TENG sensor (Reprinted with permission from ref. [64]. Copyright 2019 John Wiley & Sons); 2020s—sensors with advanced functions enabled by artificial intelligence and a triboelectric smart glove with the sign language recognition and VR space bidirectional communication (Reprinted with permission from ref. [60]. Copyright 2021 Nature). (**c**) Four different working modes for TENG.

As a supplementary and/or alternative choice to batteries, energy harvesters (EHs), which can convert various available energy sources in the ambient environment to electricity, have been proposed and studied since the 1900s, with a milestone timeline shown in Figure 1a [23,24,25,26,27]. Typically, there are four main energy sources in the environments: containing mechanical energy from movement and vibration, thermal energy from temperature variance and gradient, radiant energy from sunlight and electromagnetic waves, and biochemical energy from biochemistry such as sweat [28,29,30,31]. Moving forward, to serve as the miniature power supply integrated with MEMS sensors, the MEMS energy harvesters started to gain more attention since the 1990s [32,33,34]. Generally, MEMS energy harvesters containing the MEMS-based vibration energy harvesters (VEHs) that can transfer mechanical energy to electricity based on electrostatic [35,36,37], electromagnetic [38,39,40], and piezoelectric [41,42,43,44] principles and MEMS-based thermoelectric energy harvesters (TEHs) [45,46,47,48] that generate current from the thermal gradient. To improve their output performance, several effective approaches have been widely investigated by researchers, such as broadening the operational bandwidth through applying non-linear springs and/or stoppers and upconverting the frequency through plucking springs and multi-degree-of-freedom systems for MEMS-VEHs [49,50,51,52,53,54] and applying novel structure designs and materials for MEMS TEHs [55,56,57,58,59]. Simultaneously, plenty of MEMS sensors have been designed and become commercially available since the 1980s, as shown in Figure 1b [60,61,62,63,64,65]. The silicon-based MEMS sensors are firstly widely applied, including the MEMS accelerometers, gyroscopes, pressure sensors, etc., based on the capacitance variance generated by the micro displacement of MEMS structures and piezoresistance effect of Si and Ge [66,67,68,69,70]. With the maturation of the piezoelectric materials—such as the ferroelectric lead zirconate titanate—and the fabrication process, the piezoelectric-based MEMS sensors were also emerging in the 1990s [71,72,73]. Materials with piezoelectricity can generate electrical output due to the induction of polarized electric dipole moment by external mechanical stimuli and can also have the corresponding deformation through applying external voltages. Typical piezoelectric-based MEMS sensors include accelerometers, highly sensitive mass sensors, resonators, etc., and piezoelectric-based MEMS actuators and switches are also widely applied [74,75].

With the further development and requirement of IoT, the application scenarios of wireless sensors are not confined to buildings, machines, or vehicles, but extend to ourselves. The traditional ways of interacting with the world have been revolutionized by the flourishing of wearable and implantable devices merging with our bodies, which can monitor our motion, body temperature, respiration rate, blood pressure, heart pulse, etc. [76,77,78,79,80]. However, previous MEMS-based EHs and sensors cannot fully fulfill those new requirements. The MEMS-based EHs usually can only work in a high-frequency range or generate μW-level output power, while the human motions have low frequency and irregular patterns, and current wearable devices require at least an mW-level power supply [81,82,83,84,85]. The MEMS-based sensors, although they have high accuracy and stability and are currently only commercially available sensors, the large data volume generated will also lead to enormous power consumption in data collection, processing, and transmission [86,87].

To serve as promising platforms for self-powered sensors and simultaneously the flexible EHs for wearable and implantable devices, the piezoelectric nanogenerator (PENG) and the triboelectric nanogenerator (TENG) were proposed one after another in 2006 by Wang et al. [88] and 2012 by Fan et al. [25]. The PENG works based on piezoelectric materials with nanometer structures such as zinc oxide nanowires (NWs), which have advantages of high sensitivity, high durability, and large power density [89,90,91]. Previously, there has already been an abundance of piezoelectric materials such as single-crystal piezoelectric ceramic lead magnesium niobate-lead zirconate titanate (PMN-PZT), barium titanate (BaTiO3), zinc oxide (ZnO), poly(vinylidene fluoride) (PVDF), and its copolymer, poly(vinylidene fluoride-trifluoroethylene) (PVDF-TrFE) [73,92,93,94,95]. The piezoelectric technology also has received flourishing development and has shown remarkable performance for energy harvesting in the past few decades [96,97,98,99,100,101]. In 2006, Wang et al. firstly used zinc oxide nanowire arrays to develop the PENG for electricity generation from tiny ambient movements [88]. Typically, there are two main structures to achieve wearable textile PENG for harvesting biomechanical energy: layer stacking and yarn intersection. Layer stacking is a straightforward method with a simple fabrication process for PENG, which normally contains a piezoelectric material layer and two electrode layers, and the piezoelectric layer can be formed through vertically growing ZnO nanorods [102], PVDF nanofibers [103], PZT thin film [104], etc. However, the large thickness caused by the multilayer structure will also lead to the limitation in wearability and breathability. Therefore, the textile PENG fabricated with a yarn intersection structure based on piezoelectric fibers becomes more desirable to ensure wearable comfort. In 2008, Qin et al. fabricated the first fiber-based PENG with radially growing ZnO nanowires [105]. After that, the fiber-based textile PENG has been continuously optimized: introducing traditional fibers with PENG fibers to improve wearability and avoid short circuits [106], fabricated through other materials such as BaTiO_3_ to enhance output performance [107], applying new structures such as the 3D textile PENG to enlarge deformation and increase output [108], etc.

As another promising technology for biomechanical energy harvesting, The TENG works based on the triboelectric effect, which is a coupling effect of contact electrification and electrostatic induction caused by the potential difference of two materials [109,110,111,112]. TENG stands out for its widest material choices and simple fabrication process and has four main working modes as drawn in Figure 1c, including the vertical contact separation mode, contact-sliding mode, single-electrode mode, and freestanding mode [113,114,115]. Therefore, some optimization methods have been widely explored to further improve the energy efficiency and output of TENG, including utilizing an optimum contact structure, applying effective circuit design, and upgrading power management [116,117,118]. Liu et al. designed a bioinspired photoelectric-electromechanical integrated TENG, which can increase the surface charges through introducing the photogenerated electrons from photocatalysis and reached a current density of 221.6 μA cm^−2^ [119]. Similarly, Huang et al. applied the piezoelectric charges generated by the PCDF layer to enhance the potential difference between two triboelectric layers and significantly increase the transferred electrons in the electrification, and achieve the maximum power density of 4016 mW m^−2^ [120]. Applying Bennet’s doubler structure, which can continuously double a small initial charge through a sequence of contact-separate operations with three plates, as a charge pump is also a useful method for efficiency enhancement [121]. To overcome the air breakdown caused by the high output voltage of TENG, a buffer capacitor can be utilized, and a charge density of 1.85 mC m^−2^ has been obtained [122]. In brief, either TENG or PENG does not depend on complex MEMS fabrication suit for expansile demand of harvesting distributed energy resources among the applicational environment, which also demonstrates advantages of being cost-efficient and environment friendly. To further achieve self-powered sensors, these two technologies can generate electric power while sensing mechanical parameters such as pressure, rotation angle, vibration frequency, and so on.

Other energy harvesting technologies such as photovoltaic converting solar energy into electricity via separating semiconductor materials generated electron-hole pairs, transporting charges to electrodes, thermoelectric converting thermal energy into electricity from temperature gradients, and pyroelectric converting thermal energy into electricity from temperature fluctuations, are also chosen to support self-powered or self-sustained applications while those scenarios confront sufficient solar or thermal energy. Thus, photovoltaic self-powered gas sensors [123,124], photovoltaic self-powered photodetectors [125,126], self-powered radio-frequency sensors [127,128], photovoltaic self-powered electronic skin [129,130] have been developed by integrating photovoltaic units with related sensors. Thermoelectric self-powered wearable electronics, [131,132,133,134,135], thermoelectric self-powered electronic skin [136], and thermoelectric self-powered mercury ion sensors [137], pyroelectric self-powered breathing sensors [138,139], and pyroelectric self-powered temperature sensors [140] have been reported by utilizing thermal energy from human body or environment.

The evolution of energy harvesting technology and sensor technology has resulted in the achievement of self-powered or self-sustained features in a variety of applications. Aiming at achieving a holistic self-sustained IoT system, a large number of research with cooperation from various fields has been performed. Within this scope, research toward a highly efficient energy storage unit such as the supercapacitor [141,142] and multiple reliable self-powered sensors such as chemical sensors [143,144] and mechanical sensors [145,146] achieved by nanogenerators have been profoundly and comprehensively reviewed. However, challenges remain, such as the gap between EHs’ output and electronics power consumption, as well as effective energy utilization techniques for IoT devices. Therefore, this review will focus on introducing the system-level achievements and challenges for the self-sustained IoT system. In the following sections, solutions have been reached and reviewed in specific instances. Furthermore, typical and new applications focused on smart home, gas sensing, human monitoring, robotics, transportation, blue energy, aircraft, and aerospace, are discussed to provide an overall prospect of smart cities.

## 2. Self-Powered Sensors and Wearable Solutions

**Figure 2:**
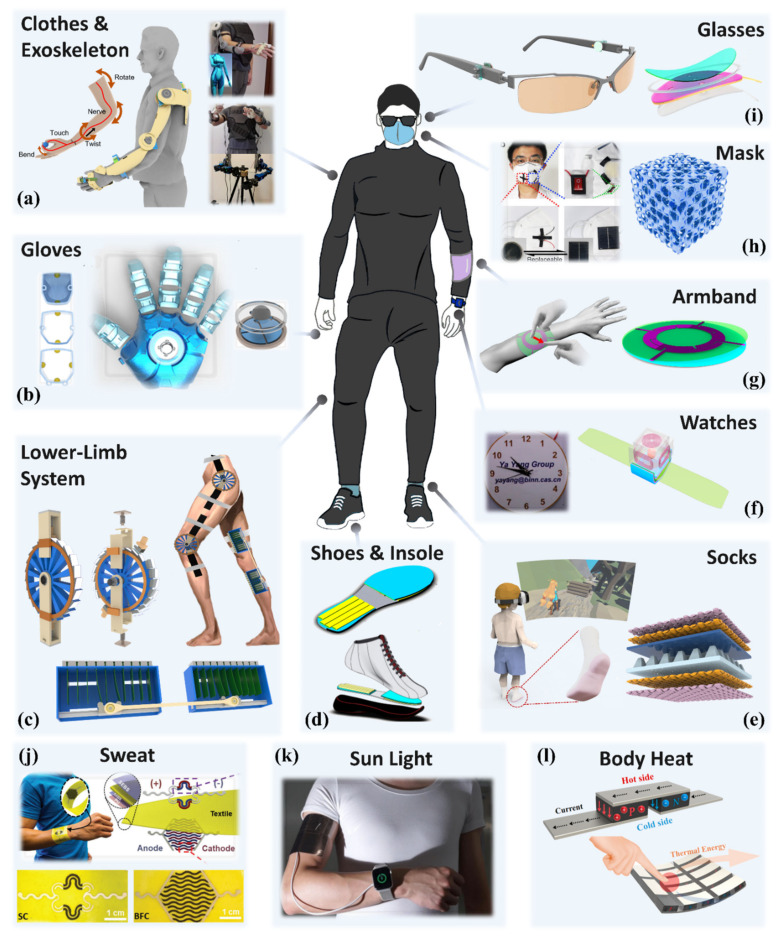
Self-powered sensor and energy harvesters: (**a**) devices applied on clothes and exoskeleton (Reprinted with permission from ref. [148]. Copyright 2021 Nature); (**b**) devices equipped on gloves (Reprinted with permission from ref. [149]. Copyright 2020 American Association for the Advancement of Science); (**c**) structures designed for lower limb (Reprinted with permission from ref. [150]. Copyright 2021 John Wiley & Sons); (**d**) devices applied in shoes and insole (Reprinted with permission from ref. [151]. Copyright 2020 American Chemical Society); (**e**) TENG-based smart socks (Reprinted with permission from ref. [152]. Copyright 2020 Nature); (**f**) hybridized energy harvester as a self-powered watch (Reprinted with permission from ref. [153]. Copyright 2015 American Chemical Society); (**g**) flexible TENG patch as an armband for human-machine interface (Reprinted with permission from ref. [154]. Copyright 2019 Elsevier); (**h**) self-powered air filter applied in a mask (Reprinted with permission from ref. [155]. Copyright 2020 Nature); (**i**) eye motion triggered self-powered sensing system equipped on glasses (Reprinted with permission from ref. [156]. Copyright 2017 American Association for the Advancement of Science); (**j**) hybrid textile devices for harvesting biochemical energy from sweat (Reprinted with permission from ref. [157]. Copyright 2018 Royal Society of Chemistry); (**k**) flexible perovskite solar cell for harvesting energy from light (Reprinted with permission from ref. [158]. Copyright 2020 Nature); (**l**) flexible thermoelectric generator for harvesting energy from body heat (Reprinted with permission from ref. [159]. Copyright 2019 American Chemical Society).

Currently, the human body can carry various wearable electronics such as smartwatches and shoes to monitor motions and health status [86], and the human body itself obtains energy sources such as thermal energy (body temperature, respiration heat, and evaporation heat), chemical energy (glucose, lactic acid, sweat), and mechanical energy (body movement, respiratory movement, and heartbeat) [147]. Thus, the EHs have been reported broadly and applied in the wearable-related energy harvesting and sensing fields, as shown in Figure 2. Some typical devices include an exoskeleton based on bidirectional TENG sensors for multiple degrees of freedom human motion sensing (Figure 2a) [148], a smart glove with TENG sensors and piezoelectric haptic feedback for human-machine interface (Figure 2b) [149], a lower-limb system with both TENG and PENG for rehabilitation and biomechanical energy harvesting (Figure 2c) [150], an insole based on TENG for scavenging the unused energy in walking (Figure 2d) [151], a smart sock that can monitor the human motion status and distinguish user identity with the output of TENG (Figure 2e) [152], a self-powered watch with the energy captured by TENG and electromagnetic generator (EMG) from human motions (Figure 2f) [153], a multi-functional armband based on TENG with grid-patterned electrodes for wireless communication and vehicle manipulation (Figure 2g) [154], a self-powered air filter applied in the mask (Figure 2h) [155], and a self-powered mechanosensation communication system based on TENG utilizing the eye motions (Figure 2i) [156], a sweat-based hybrid textile devices with biochemical energy harvesting from sweat and energy storage (Figure 2j) [157], a wearable perovskite solar cell for scavenging infrared energy from light (Figure 2k) [158], and a hybridized thermo-triboelectric generator for utilizing both mechanical energy and thermal energy from body motions and body heat (Figure 2l) [159].

Based on the preceding examples, self-powered sensors have been developed as supplementary elements that do not place a heavy load on users. After studying technical parameters such as sensitivity and range of linearity about pressure, strain, humidity and temperature, responsivity to light or temperature, the detection limit of targeted gas, and durability for continuously working, developed self-powered sensors have demonstrated considerable capabilities of commercial sensors. Additionally, by incorporating EHs into commercial products such as gloves, shoes, and exoskeletons, limbs with big amplitude motions have received a lot of attention. Furthermore, the hybridized mechanism comprising several energy harvesting technologies is used to bridge the gap between energy generation and power consumption. The self-powered sensors, which are intended to monitor eye movements, respirations, and face movements, are lightweight and small in size, with a priority on precise sensing via multiple sensors and advanced data analysis. A self-powered body sensor network can be envisaged in future research and production through the conjunction of EHs and sensors.

## 3. Self-Sustained IoT System and Emerged Approaches

**Figure 3:**
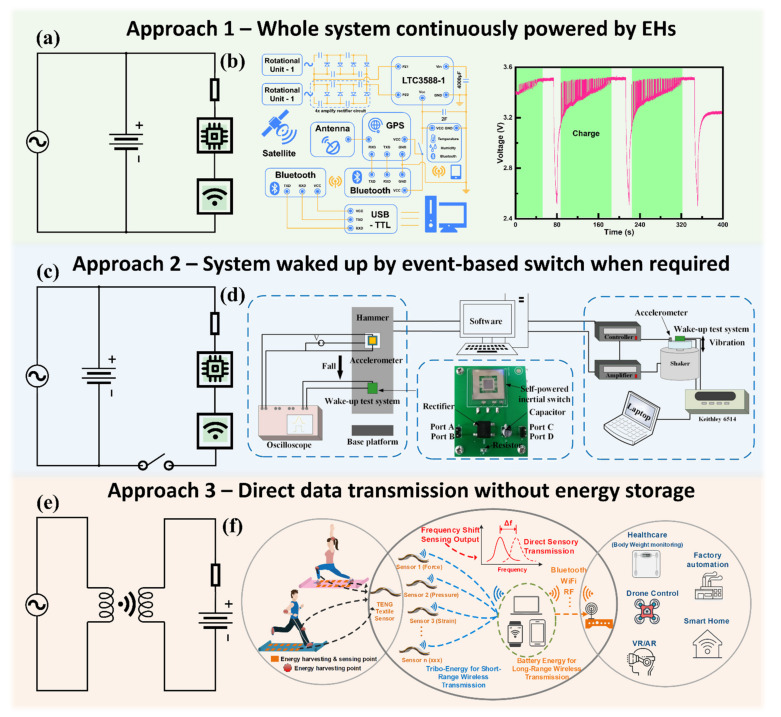
Three Approaches Toward Self-Sustainable IoT System: (**a**,**b**) approach 1—whole system continuously powered by EHs, (Reprinted with permission from ref. [166]. Copyright 2021 American Chemical Society); (**c**,**d**) approach 2—system waked up by event-based switch when required, (Reprinted with permission from ref. [167]. Copyright 2021 IEEE); (**e**,**f**) approach 3—direct data transmission without energy storage (Reprinted with permission from ref. [168]. Copyright 2020 Elsevier).

Combining the wireless self-powered sensors and EHs, IoT systems with self-sustainability have been successfully achieved and applied in urban and natural environments [160,161,162]. Recent works include a wristband with self-sustained gas sensing, an armband for electrocardiography, and wearable textile for rehabilitation [163,164,165]. Here, we divide the self-sustainable IoT systems into three categories based on their different approaches, as shown in Figure 3.

The first approach is also the most typical approach for current self-sustainable IoT systems, with a schematic circuit drawn in Figure 3a. The energy harvesters will firstly charge the energy storage unit such as a supercapacitor, and the supercapacitor will keep powering all of the functional units in this system, including sensors, processors, and wireless data transmission modules. A typical example is shown in Figure 3b [166]. The ultra-low frequency biomechanical energy harvested by two rotational units equipped on a walking stick can power an IoT system with a GPS module, an environment amenity sensing module, and a wireless module. However, that always-on system requires a high-power supply, which is not always feasible. For current commercialized IoT systems, typical sensors such as accelerometers have a power consumption ranging from 100 μW to 1 mW in normal operation mode. With this power consumption, a typical button cell battery with 50 mAh capacity will only last a few days, and the lifetime will be even shorter with a wireless communication module.

With the growing applications in IoT, commercial sensors have also begun to include low power or wake-up modes, where the sensor normally works with a significantly lower sample rate, and only jumps back to its normal operation mode when a sufficiently large signal is detected. Although such methods can reduce the power consumption, it still needs external circuits to analyze the data from sensors, which requires extra power supplies. Typically, the power consumption of such systems can be reduced to around 10 μW, a power consumption that allows the thin-film batteries to only last a day at best. To solve this problem and significantly reduce the system power consumption, the second approach was proposed, which introduced a zero-power event-based switch, as shown in Figure 3c. The system is mainly in the sleep mode, in which the energy harvesters can keep collecting energy from ambient environments and storing it in the energy storage unit, while the switch keeps open and does not power other units. Only when specific events happen, which exceed a certain threshold and let the event-based switch become close, the functional units will be powered and send out the critical obtained sensing data. The example is shown in Figure 3d [167]. The inertial switch serves as a MEMS-VEH under acceleration smaller than its threshold. Under an emergent situation (hammer falling), the acceleration applied to the inertial switch will become higher than the threshold and trigger the system wake-up. In these two approaches, an energy storage unit (super capacitor or battery) as the reliable power supply and a wireless communication module to send out sensing data are indispensable parts, which inevitably lead to system complexity, large size, and high-power consumption. Therefore, the third approach was proposed based on the direct wireless transmission of the voltage signals generated by self-powered sensors. Through inductive coupling, the sensing output can be directly sent out without the requirement of energy harvesters, energy storage units, and wireless modules, as depicted in Figure 3e. The example is shown in Figure 3f [168]. The output generated by textile TENG arrays can be directly sent to the data processing circuit through the induct coupling of coils with a maximum transmission range of 1 m. The corresponding sensing information—such as the force, strain, and pressure—can be obtained through analyzing the frequency shift. Therefore, no battery or other energy storage units are required for the wireless sensing node. However, although with a simple system and low power consumption, approach 3 also has the limitation in the data volume and communicating range. The choices in those three different approaches should be made considering specific application scenarios. For the applications requiring continuously high-resolution sensing data such as building monitoring, approach 1 can be applied with designed energy harvesters to extend the battery life and self-powered sensors to reduce the power consumption. For the applications aiming for recording specific events such as falling, approach 2 will be a better choice for a near-zero-power system with an event-based switch. For the applications with simple monitoring data and require the most compact system design, such as some wearable and implantable devices to monitor the heart rate or body temperature, approach 3 which only contains the self-powered sensor and coils can take lead. The achievement of self-sustainability will greatly help to further improve the feasibility of equipping wireless sensing nodes in various environments, and more diversified IoT applications will become achievable.

## 4. New Applications in Different Scenarios

### 4.1. Smart Homes

**Figure 4:**
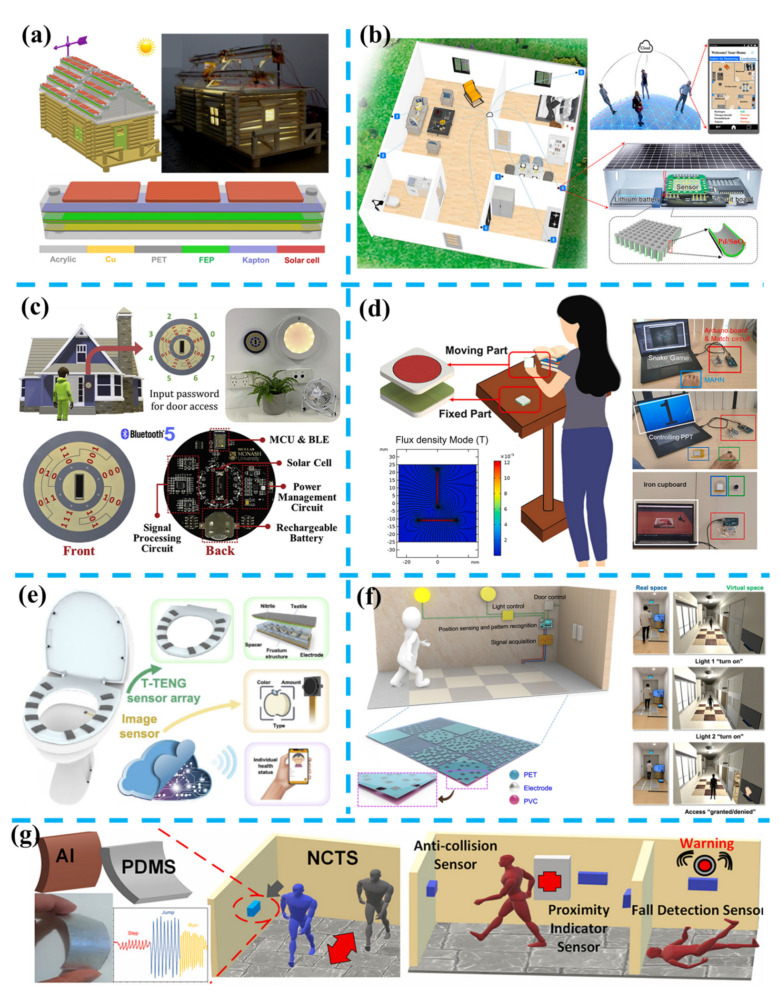
Smart home. (**a**) Roof for scavenging solar and wind energies (Reprinted with permission from ref. [170]. Copyright 2016 American Chemical Society). (**b**) Wireless self-powered high-performance integrated nanostructured-gas-sensor network (Reprinted with permission from ref. [174]. Copyright 2021 American Chemical Society). (**c**) Self-powered control interface for accessing based on Gray code with hybrid triboelectric and photovoltaics energy harvesting (Reprinted with permission from ref. [176]. Copyright 2020 Elsevier). (**d**) Noncontact control interface based on magnetic-interaction assisted hybridized triboelectric-electromagnetic nanogenerator (Reprinted with permission from ref. [177]. Copyright 2021 Elsevier). (**e**) AI-Toilet for an integrated health monitoring system (IHMS) using smart triboelectric pressure sensors and an image sensor (Reprinted with permission from ref. [178]. Copyright 2021 Elsevier). (**f**) Smart mats as a scalable floor monitoring system enabled by deep learning (Reprinted with permission from ref. [8]. Copyright 2020, Nature). (**g**) Contactless tracking of humans in a smart home using non-contact triboelectric sensing technology (Reprinted with permission from ref. [179]. Copyright 2021 Elsevier).

To build an integrated and energy-efficient smart home’ system, energy sources among indoor environment and outdoor environment can be utilized to address the power consumption of sensor nodes. As for the roof, which possesses features of relatively large area and facing solar, wind and rain. In 2015, Zhang et al. introduced lawn structured TENGs for scavenging sweeping wind energy on rooftops and were capable of lighting 60 LED [169]. Furthermore, Wang et al. reported a strategy of energy harvesting with a hybridized nanogenerator [170], as shown in Figure 4a. A single device included three panels of Si-based solar cells on the top and a flutter structural TENG under solar cells. Thus, the device can scavenge solar and wind energies when installed on the roofs, and results have shown the superiority of charging the Li-ion battery by using a hybridized strategy than an individual strategy. To harvest rain energies, Liu et al. prepared a Si-based solar cell with a mutual electrode (PEDOT: PSS) of a single-electrode-mode TENG placed on the top [171]. Similarly, Yoo et al. prepared a moth’s eye mimicking TENG on the solar cell to reduce the side effect from the triboelectric layer by its excellent specular transmittance [172]. Additionally, Zhao et al. demonstrated a TENG/Si tandem hybrid solar cell by stacking Ag/PDMS electrode with a Si-based solar cell, in which both photovoltaic and triboelectric performances were enhanced by increased light transmittance and build-in electric field [173]. As a result, this tandem hybrid solar cell obtained a higher power conversion efficiency of 22.04% than the pristine solar cell. In brief, solar energy, wind energy, and rain energy, all can be converted into electricity and promised to power sensor nodes in smart home applications. In Figure 4b, Song et al. demonstrated a realistically available example for smart homes, titled as a wireless self-powered integrated nanostructured-gas-sensor network (SINGOR) for smart homes [174]. This SINGOR system consists of a power supply module, gas-sensing module, microcontroller-analog readout circuit, and wireless data transmission module, and Song et al. had optimized each mode to obtain self-powered ability. First, rational power management was applied to adjust strategies of indoor light’s energy harvesting and lithium-ion battery’s charging, resulting in an intermittent mode that was activated to extract power from the solar cell panel and charge the battery as the indoor light was on, or disable the charging function when lacking sufficient indoor light illumination. Second, a 4 × 4 sensor array with an ultralow-power consumption of 68.6 μW was integrated with a readout printed circuit board. Third, a microcontroller and a Bluetooth unit were applied for data acquisition, analog−digital conversion and wireless transmission, and the average power consumption was further lowered down by different duty cycles of the intermittent mode which included on-cycles of continuous sensing, and off-cycles of sleep mode and shut-down of the Bluetooth unit. In their demonstration, four SINGOR nodes were deployed at the corners of a kitchen and formed a smart gas sensing network, the gas leakage alarming and source location of H_2_ were achieved and displayed on a mobile phone. It is worth mentioning that mechanical energy among indoor environment also can be utilized to realize monitoring in smart homes, and Graham et al. fabricated a series of contact-separation mode TENGs with plastic and electronic waste, and placed them in different regions of the home to harvest mechanical energy from daily human activities [175], which can also monitor interactions between human and machines among smart homes.

Regarding the abovementioned interactions such as touching and pressing, as shown in Figure 4c, Qiu et al. reported a self-powered control interface for accessing based on Gray code with hybrid triboelectric and photovoltaics energy harvesting [176]. This device consists of a sliding operation TENG with three kinds of underneath electrodes and a solar cell; thus, it can not only harvest indoor light energy but also mechanical energy via hand tapping. With the application of a microcontroller with a BLE 5.0 module, sliding motion cross different electrodes can be converted into 3-bit binary-reflected Gray-code (BRGC) and achieve wireless control of smart appliances, and a sleep mode will be activated after 5 s idle state to save power. Additionally, power consumption in a controlling operation can be offset by 55 s of harvesting indoor lighting, and power consumption in sleep mode for 120 s can be recovered by harvesting mechanical energy by 40 s of hand tapping. Based on the 3-bit code generated by eight sliding directions, the device is applied to build a home authentication access control system, which requests the user input 6-digit password by sliding on the disk interface. Compared with the former integrated device, He et al. reported a triboelectric vibration sensor (TVS) for establishing a human-machine interface on ubiquitous surfaces [180], which mimics the cilia wrapped in the ampulla structure in the fish’s lateral line and fabricate hierarchical structures with interlocked polyethylene (PE) nanowire arrays and hemispherical pores. Based on its high force sensitivity of 0.97 V/N, three TVS are utilized to identify keystroke positions in an intelligent virtual numeric keyboard according to the time lags at the highest peaks of the cross-correlation functions. The keystroke dynamics of different users are further extracted to build a profile database and trained to find the authentic user from the same keystroke sequence. Moreover, a multifunctional desktop interactive system is developed by positioning the tapping area to control lights, fans, and speakers wirelessly, which demonstrates as a facile control interface in smart homes. The noncontact/touchless control interface is another noteworthy application, and researchers have developed various interfaces based on humidity sensing [181,182], capacitive sensing [183,184], magnetic sensing [185,186], and triboelectric sensing [179,187,188]. As shown in Figure 4d, Liu et al. introduced a magnetic-interaction-assisted hybridized triboelectric-electromagnetic nanogenerator (MAHN) to achieve noncontact control and recognize simple air gestures [177]. The MAHN consists of the moving part and the fixed part, in which a pair of attracted magnets are respectively embedded in each part, thus two parts are attracted to each other at the rest state. As the user lifts the moving part, hold it in the vertical plane, and swing it above the fixed part, the electromagnetic part in the MAHN will generate different voltage curves from different swing direction. In the demonstration of controlling PowerPoint document, operations of lifting, putting down, swing from left to right, and swing from right to left correspond to full screen, end presentation, page down, and page up, which realizes most functions of the commercial laser pointer but with a self-powered design. Moreover, the TENG part with four electrodes can also map moving directions of up, down, left and right into a “snake game”, via detecting positive peaks in four channels. Beside applying signals from hybridized mechanism, the MAHN as a hybridized energy harvester can power a thermometer or a Bluetooth module by converting mechanical energy from strong magnetic attraction. From above illustrations, control interface developed from energy harvesting technologies in smart home not only can transfer information to machine, but also replenish electric power from human-machine interactions.

As shown in Figure 4e, Zhang et al. proposed an AI-Toilet for an integrated health monitoring system (IHMS) using smart triboelectric pressure sensors and an image sensor [178]. On the basis of a commercial toilet, 10 textile-based TENGs are attached to the toilet seat to collect pressure data with a microcontroller unit from different users, which offer a more private approach with the advantages of low cost and easy fabrication. With the aids of deep learning, the user seating on the toilet seat is identified from a database of 6 volunteers with an accuracy of 90%. Based on the identification in privacy, additional information such as seating time and urine color can be recorded by the TENGs and image sensors for health assessment. Other usual facilities in smart homes such as the floor/mat are also worthy improved by energy harvesting technology. Kim et al. applied piezoelectric-based floor tile to power a wirelessly transmit node and control an air conditioner, a standing lamp, or an air purifier, and that prepared floor tile shows good stability for 30 days in responding to the user’s weight [189]. Gu et al. introduced cellulose-based TENGs as an eco-friendly energy harvesting floor, which achieve a 1:1 footstep-to-signal (transmitted and received) ratio in this build wireless transmission sensing system [190].

For unexpected hazards, Ma et al. reported a self-powered escape and rescue system with flame-retardant single-electrode triboelectric yarns (FRTY) spun by a continuous and scalable production spinning technology [191]. In the demonstration, the 3D honeycomb-structured fabric TENGs woven from the FRTY are integrated into smart carpets, and victims in the fire can send accurate real-time location through tapping the nearest carpet, and electric power generated by every step on the carpet can light arrow guidance in the shortest escape path. Dong et al. reported another fabric TENGs as e-textiles for power and sense, which are flexible, shape adaptable, and cyclic washable [192]. Benefit with the integrated 3D braided structure, the e-textiles have no significant reduction of outputs during one month of cyclic loading and after 20 times washing. Furthermore, a self-powered identity recognition carpet is demonstrated with 128 equally sized square blocks where the 3D braided TENG fabrics are sewn at the back center of the 64 functional black blocks and play as mutually independent sensing regions linked to corresponded synchronous acquisition channels. Real-time walking trajectories from different users are recorded and utilized to verify if it is matched with the set password path. By unitizing AI technologies, as shown in Figure 4f, Shi et al. developed a scalable floor monitoring system for indoor positioning, activity monitoring, and individual recognition toward the smart building/home application. Specifically, six kinds of mats with different electrode coverage rates (from 0% to 100%) with a 20% difference are fabricated by low-cost and highly scalable screen-printing technique and formed a 3 × 4 mat array with parallel connection. In the demonstration in virtual space, the position of each step in real space is adopted for the light control at the corresponding sites, and the full walking signal sent to the CNN model is utilized to identify the registered users for door access auto-control. The smart floor monitoring system produces a high prediction accuracy of 96% of a 10-person model, offering a highly secure, convenient, and accurate approach for individual recognition, which is superior to the camera and smart tag-based individual recognition. Recently, as shown in Figure 4g, Anaya et al. applied non-contact triboelectric sensing technology to track human motions in smart homes [179], in which the flexible non-contact triboelectric sensors (NCTS) are applied to detecting dynamic external electrostatic charges from human’s walking, running, jumping and relative motions of two people within a distance of 1.5 m. Furthermore, a portable accident prevention system integrated with two sensors is developed to avoid visually impaired people’s collisions with obstacles and the elderly’s unexpected falling. In brief, smart homes integrated with self-powered sensors and self-sustainable IoT systems not only improve the interactivity between humans and machines but also are favorable enhancing humanization and security towards all kinds of users.

Through the above examples related to energy harvesting, self-powered sensing, and self-sustained IoT monitoring, researchers have surveyed solutions from materials fabrication, circuit design, and signal processing. However, the smart home is an integrated concept from build to decorate, in which few works have been discussed with self-powered and self-sustained designs. The walls confronting illumination, sunlight, and temperature variations can be powerful energy harvesting units to support sensing and IoT applications in smart homes. Moreover, conditioner and windows linked indoor environment and outdoor environment can be realized self-powered sensing by extracting energy and information from airflow and lighting. The intelligent control among different interfaces is also needed to wirelessly connect through wireless self-powered nodes, which are potentially powered with harvested energy from interactive operations.

### 4.2. Gas Sensing

Gas sensing, targeting environmental detection [193,194], healthcare analysis [195,196], and security monitoring [197,198], is a critical infrastructure in smart home and city construction. Current rapid advances of the internet of things and wearable devices also demand new self-powered or zero-power gas sensing nodes. Several approaches, including triboelectric nanogenerators [199,200,201], passive photonic platforms [202,203], zero-power 2D material-based photodetectors [204,205], have been extensively explored in the past few decades to enable this battery-free property.

**Figure 5:**
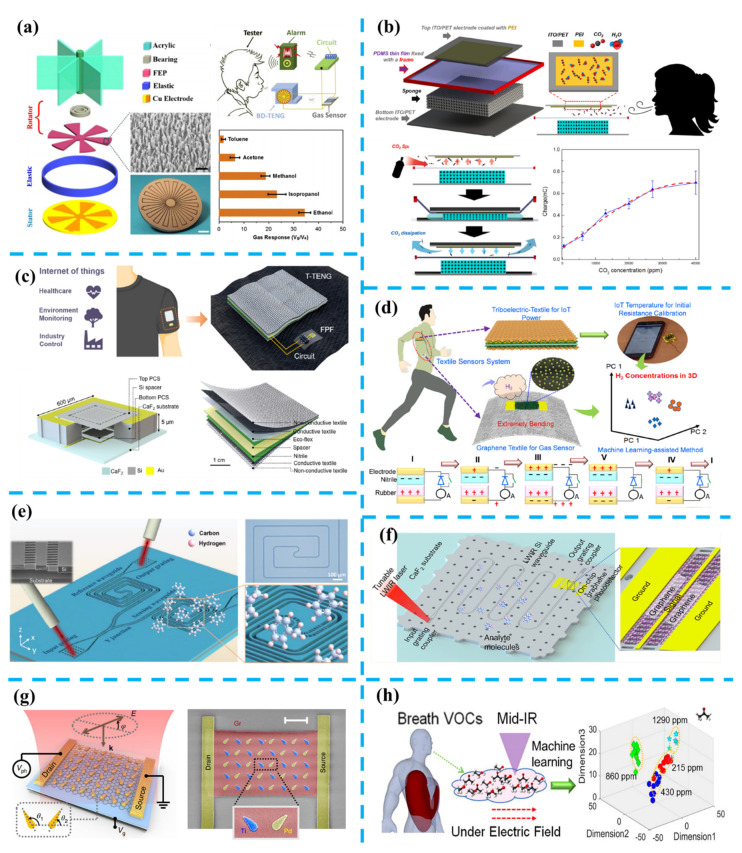
Gas sensing for smart home applications. (**a**) Blow-driven alcohol breath analyzer (Reprinted with permission from ref. [207]. Copyright 2015 Elsevier). (**b**) Self-powered amenity sensor based on the water-air triboelectric nanogenerator (Reprinted with permission from ref. [208]. Copyright 2017 American Chemical Society). (**c**) Triboelectric-enabled wearable mid-infrared computational spectrometer for gas sensing applications (Reprinted with permission from ref. [209]. Copyright 2021 Elsevier). (**d**) Textile-based graphene gas sensor with energy harvester (Reprinted with permission from ref. [210]. Copyright 2021 Elsevier). (**e**) Toluene monitoring for environmental and healthcare [197]. (**f**) Graphene spectrometer for sensing applications (Reprinted with permission from ref. [204]. Copyright 2021 American Chemical Society). (**g**) Zero-bias graphene polarization detector for sensing applications (Reprinted with permission from ref. [211]. Copyright 2021 Nature). (**h**) VOC identification toward healthcare diagnoses (Reprinted with permission from ref. [195]. Copyright 2021 American Chemical Society).

Triboelectric nanogenerators (TENGs), representing a thriving renewable energy technology and can function as energy harvesters [7,206], have the potential to free the gas sensor from the heavy battery requirement. From the perspective of the framework, TENG-based sensors can be divided into two major groups. One is using TENG as both the power source and sensor node, making the TENG sensor a self-powered system. The other approach is combining TENG as an energy harvester and a sensor with a different principle. In 2015, Wen et al. proposed a blow-driven alcohol breath analyzer fabricated by the TENG, with the capability to detect alcohol selectively [207]. As shown in Figure 5a, the blow-driven triboelectric nanogenerator (BD-TENG) consists of three parts: a rotator blade that convert the blow into the mechanical rotation with an acrylic disk and fluorinated ethylene propylene (FEP) top layer; a stator composed of two copper (Cu) electrodes; a soft elastic made of sponge that acts as a spacer between the FEP layer and the Cu thin film. During the breath, the air blowing will generate electricity from the rotational motion of the blade, which is functioned as the power source of the system. The contained alcohol will be absorbed with a resistance change of the FEP polymer nanowires, which is worked as the sensing part and are electrically driven by the rotational TENG. This BD-TENG features a high detection gas response of ~34 and a fast response time of 11 s. This sensing system experimentally achieves a 10 ppm limit of alcohol vapor detection, with an outstanding selectivity over other chemicals such as isopropanol, methanol, acetone, and toluene.

In 2017, Wang et al. proposed a water-air triboelectric nanogenerator (WATENG) targeting the self-powered amenity sensor [208]. As depicted in Figure 5b, the WATENG is comprised of a polyethyleneimine (PEI) adsorptive layer with the interference of humidity. The charge curve will vary with different CO_2_ concentrations and relative humidity (RH). In this work, the RH and the CO_2_ concentration can be determined separately. The effect of the RH on triboelectric output is well studied and completely calibrated in the measurement of CO_2_, making this WATENG can be used for CO_2_ sensing for environments with different RH. With such a calibration strategy and PEI adsorptive coating, the sensing range can be up to 30,000 ppm with a detection limit of 420 ppm. Another approach that applies TENGs as power supplies for the rear-end gas sensor nodes is also well explored. In Figure 5c, Chang et al. leveraged textile-TENG (T-TENG) to dynamically tune Fabry-Pérot (FP) photonic crystal slab (PCS) filter, demonstrating this system as a wearable mid-infrared computational spectrometer for gas sensing applications [209]. The FP filter consists of top silicon PCS, air spacer, and bottom Si PCS. Its reflectivity is enabled by the guided resonance. The central wavelength will shift with the change of the distance between the top and bottom Si PCS. This FP filter can be treated as a capacitor and can take advantage of the high open-circuit voltage output from T-TENG. The whole optical spectrum can be reconstructed from the different statuses of the FP filter, which is altered by the output voltage of the T-TENG. The feasibility of this wearable computational spectrometer is well proved by taking CO_2_ and acetone sensing as demonstrations at the wavelength range of 4–5 μm and 5–6.5 μm, respectively. In 2021, Zhu et al. leveraged the energy harvester and machine learning to enable a robust wearable hydrogen sensor for IoT applications [210]. The sensing mechanism is based on the catalytic effect of palladium nanoparticles on the wide bandgap-reduced graphene oxide (Figure 5d). This graphene textile gas sensor shows a six times higher sensing response than the graphene polyimide membrane gas sensor due to its large surface area.

As passive platforms, optical gas sensors can be externally probed by laser diodes and, as a result, work as a zero-power sensor node. Absorptive optical sensors have the advantage of label-free analysis capability, arising from the unique characteristic spectrum of a given molecular species [212,213]. This property makes the identification and detection of a complex mixture of different molecules feasible with a straightforward approach. Optical waveguide-based platforms provide an attractive way for sensor miniaturization and integration with other optoelectronic circuits [214,215,216,217]. In 2021, Liu et al. proposed a suspended silicon waveguide platform working at a mid-infrared regime for toluene sensing [197]. As depicted in Figure 5e, the waveguide is probed by optical fibers, and the light intensity will reflect the concentration of the toluene. With an optimized waveguide structure, a low detection limit of only 75 ppm is experimentally achieved with a fast response time of only 0.8 s. This platform is envisaged to offer a pathway toward the realization of high-performance chip-scale optical sensors for real-time environmental and medical applications. Above this, Ma et al. from the same group integrated graphene photodetectors to the waveguides [204]. As depicted in Figure 5f, the integration of waveguides and photodetectors is an indispensable step toward the realization of optical sensing systems with a minimized power consumption and footprint [205,218]. Among numerous 2-D materials that have been demonstrated as photodetectors, graphene has a zero bandgap, which enables broadband responsivity and zero-bias operation. This property makes graphene can be used as an almost zero-power photodetector. In Ma’s work, ~8 mA/W responsivities are achieved in the low long-wave infrared wavelength range of 6.3–7.1 μm, and detection of 0.72% toluene is demonstrated based on this platform. Optical sensing platforms can distinguish not only the specific gas composition but also the unique properties of molecules such as chirality [219,220,221]. This is attributed to the synchronization between optical polarization and chiral molecular symmetry. Exited by the light with different polarization, specific molecules with chirality will respond differently. This property enables us to differentiate chiral molecules in the optical domain directly. Therefore, on-chip polarization-sensitive photodetectors offer unique opportunities to detect chiral molecules such as glucose [222]. In 2021, Wei et al. proposed a mid-infrared graphene detector with polarization-detection capability [211], as shown in Figure 5g. The nanoantenna is patterned on top of the graphene detector to enable a configurable polarization sensitivity. This work experimentally achieves a polarization ratio as high as 100 with the polarization-angle perturbation down to 0.02° Hz^−1/2^ in the mid-infrared range. Multi-components sensing capabilities of optical sensors and their applications in environmental monitoring and healthcare are also well demonstrated over the past several years [223,224,225]. In 2020, Zhou et al. proposed a surface-enhanced infrared absorption platform enabling simultaneous on-chip sensing of greenhouse gases, including CO_2_ and CH_4_ [193]. The metamaterial absorber is periodically patterned to enable surface-enhanced infrared absorption. On top of the absorber, a metal-organic framework (MOF) film is coated, functioning as a multi gas-selective material due to its unique structural characteristics and high surface area. The simultaneous sensing of CO_2_ and CH_4_ is demonstrated with a detection limit of 1.1% for CO_2_ and 0.4% for CH_4_. In 2021, Zhu et al. proposed a machine learning-assisted plasma-enhanced infrared absorption (PEIRA) platform targeting volatile organic compound (VOC) identification and healthcare diagnoses (Figure 5h) [195]. By leveraging the high voltage generated by TENG, the authors claim the plasma discharges will be generated in the various VOC environments, and as a result, enhance the light-matter interaction. In their demonstration, methanol, ethanol, and acetone are characterized under both individual component and mixed component environments. By leveraging the principal component analysis, the identification accuracy of a specific component in the mixture is enhanced. Furthermore, the authors also did the alcohol diagnosis and diabetes identification based on this platform.

As an essential sensor type as well as a major power consumer of an IoT node, the gas sensor may significantly influence the lifetime of the whole system. The above-discussed demonstrations of battery-less gas sensors provide a promising solution to ease this constraint. Nevertheless, we also have to acknowledge that the integration of these sensor nodes to the whole IoT system is still in its infancy. There are still many problems to be tackled for the optimized performance and network deployments, such as the circuit design and signal transmission. Moreover, it is also important for most gas sensors to distinguish one or several specific gases in a complex environment. Luckily, the photonic gas sensor has the intrinsic molecular information of a given species (a.k.a. molecular fingerprint). Therefore, it may play an important role in those applications where multiple gases are involved.

### 4.3. Human Monitoring

**Figure 6:**
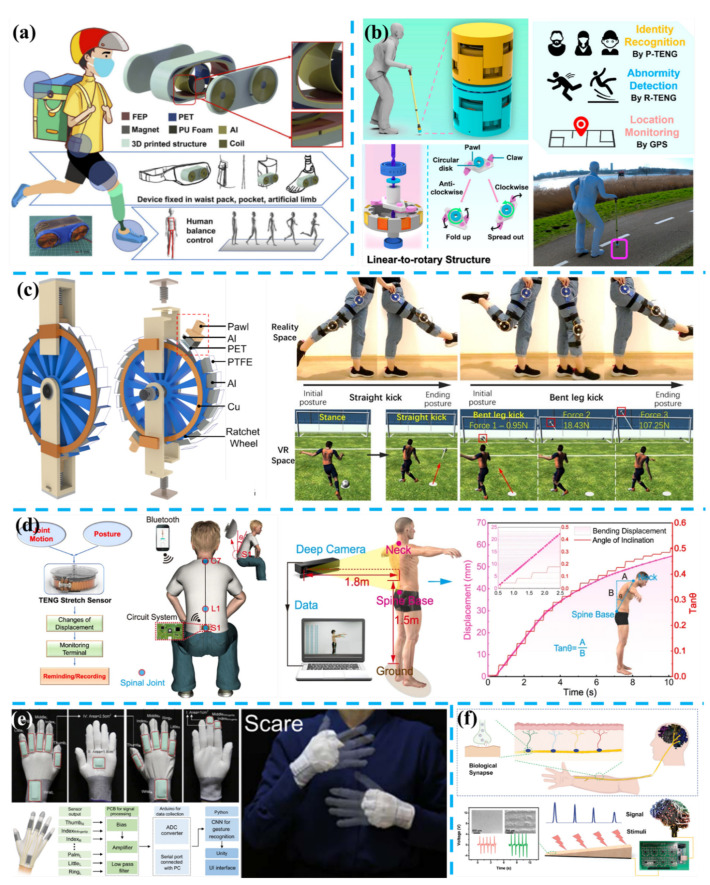
Human monitoring. (**a**) A hybrid biomechanical energy harvester designed for human balance control processes (Reprinted with permission from ref. [238]. Copyright 2021 Springer). (**b**) Artificial Intelligence-enabled caregiving walking stick for the aged (Reprinted with permission from ref. [166]. Copyright 2021 American Chemical Society). (**c**) A motion capturing and energy harvesting hybridized lower-limb system for rehabilitation and sports applications (Reprinted with permission from ref. [150]. Copyright 2021 John Wiley & Sons). (**d**) Sensing of joint and spinal bending or stretching via a retractable and wearable badge reel (Reprinted with permission from ref. [240]. Copyright 2021 Nature). (**e**) AI enabled sign language recognition and VR space bidirectional communication using triboelectric smart gloves (Reprinted with permission from ref. [60]. Copyright 2021 Nature). (**f**) TENG-based artificial perception and transmission nerve (Reprinted with permission from ref. [241]. Copyright 2021 John Wiley & Sons).

Human monitoring is undoubtedly another crucial scenario for energy harvesting technology, which is promising evolved as self-powered or self-sustainable wearable/implantable systems for movement monitoring or therapy treatment. Corresponding prototypes can be clothes [226,227,228]; footwear such as socks [152,229], insoles [151,230] and shoes [231,232]; accessories such as glasses [156], wristbands [233,234], gloves [149,215,235], and patches [131,236,237]. Liu et al. introduced a hybridized electromagnetic-triboelectric nanogenerator (HETNG) to extract energy and information from the inherent balance control processes, as shown in Figure 6a [238]. The HETNG is composed of an asymmetrical pendulum structure and a cylinder magnet rolling inside. Along with body movements induced by lifting the foot up and down during walking, the magnet inside the symmetrical pendulum structure will roll with the trunk, thus body rotations of turning left and right, and actions of stepping up and down a platform are monitored and discriminated from overlapped voltage signals of different EMGs and the middle TENG. The HETNG has also been simulated for being integrated into artificial limb by being placed on thigh and foot, and actions of squat and stand up, lifting the leg up and down are detected, and harvested biomechanical energy can power a Bluetooth module to send temperature/humidity data to a mobile phone. For the elder that walks slowly with a walking aid, the HETNG equipped on the walking aid can help to record the motions of forwarding and unexpected falling, which is useful for calling for help. In Figure 6b, Guo et al. reported an AI-enabled caregiving walking stick for elderly and motion-impaired people, by combining a linear-to-rotary structure with hybridized electromagnetic-triboelectric nanogenerators [166]. First, there are three energy harvesting parts including top press TENG (P-TENG), rotational EMG, and rotational TENG (R-TENG) in the hybridized unit, and their maximum peak power reaches 2.98 mW, 6.22 mW, and 61.4 μW at 35 MΩ, 70 Ω, and 37.5 MΩ, respectively. Second, signals collected from P-TENG are utilized to achieve contact point sensing and gait abnormality detection via deep-learning analysis approach and obtain multiple advanced functionalities including identity recognition (accuracy: 99.5%), mobility disability evaluation (accuracy: 100%), and motion status determination (accuracy: 100%). Third, in the indoor environment, the motions of users can be recognized instantaneously, and an alarm will be sent out immediately as a gait abnormality is detected; in the outdoor environment, an additional GPS sensor is self-sustained with two rotational units and applied to track the location.

Recently, as shown in Figure 6c, Gao et al. developed a motion capturing and energy harvesting hybridized lower-limb system for rehabilitation and sports applications [150]. That system consists of a sliding block-rail piezoelectric generator (S-PEG) and lower-limb motion sensing with a ratchet-based triboelectric nanogenerator (R-TENG). Thereinto, the R-TENG can capture the lower-limb posture via triboelectric signals generated from relative movements of two pawls as the leg’s lifting up and down, which match the rotation angles to detected pulse numbers. Based on the above mechanism, three imitated gait features of slightly abnormal, highly abnormal, and reduced strength are detected, and freezing of gait (FOG) in typical Parkinsonian gait is also detected to prevent potential harm after a loss of stride period. In addition, sports training programs in virtual space are demonstrated by calculating kicking force from four channels’ triboelectric signals in two R-TENG. The S-PEG with 20 bimorphs is utilized to leverage kinetic energy from lower-limb activity, resulting in high power of 2.4 mW under a very low operation frequency of 0.75 Hz, and charging a lithium battery of 350 mAh. Charging lithium batteries with energy harvesting technology is also an efficient indirect strategy for developing self-sustainable healthcare electronics [231,239]. For human body monitoring, Zhu et al. integrated triboelectric bidirectional sensors into an exoskeleton manipulator to monitor multiple movable joints of upper limbs [148]. Benefiting from the design of a bistable switch with two electrodes on the shaft and grating electrodes on the fly ring, rotational or linear motions from different joints can be converted into rotations of motors in 3D printed exoskeleton arms. Then clockwise and counterclockwise rotation of motors can both be detected by prepared sensors and further applied in kinetic analysis physical parameters such as displacement, velocity, force, etc. Eventually, virtual training demonstrations of ping-pong program and punch program are realized real-time processing by equipping this exoskeleton.

In Figure 6d, Li et al. reported a retractable and wearable badge reel-based sensor with a grating structured TENG for sensing the joint’s and spinal’s bending or stretching [240]. Compared above mechanical structures that rely on limbs’ motions, this sensor has a light weight of 9.6 g, a diameter of 33 mm, and a thickness of 10 mm. As the rotor with coil springs is rotating and stretching with the rope’s fixed end attached to the thoracic, lumbar, or sacrum segments of the spine, the stator with two groups of complementary grating electrodes will generate electric signals according to freestanding TENG. The results exhibit a stretching sensitivity of 8 V/mm and a minimum resolution of 0.6 mm. In the demonstration, a spinal monitoring system is developed, and real-time sends wireless alarm information according to customized settings, presenting superior feasibility and precision than a commercial inclinometer and a depth camera.

Another potential significative application is developing an intelligent and feasible system for disabled users losing vision or speech. Guo et al. reported a self-powered triboelectric auditory sensor (TAS) for hearing aid and enabled the hearing-impaired user to fully hear music with a low-cost prototype [242]. Qu et al. fabricated a dielectric elastomer-based refreshable Braille device actuated by a sliding type TENG [243]. The combined effect of high voltage generated by the TENG, and air pressure supported by the chamber provides the device a better tactile sensation as a Braille dot. Then Braille letters of “T,” “E,” “N” and “G” are displayed by the six devices controlled by TENGs array and MCU with different programs. Ha et al. reported a skin-inspired triboelectric sensor with interlocked and micro-ridge structured polymer architectures based on stiff P(VDF-TrFE) layer and soft PDMS layer [244]. This sensor is capable of detecting human vital signs of minute arterial pulse waves and recognition of human voice for the biometric security system. In addition, a smart glove, consisting of 11 sensors attached on the joints of fingers and wrist, is fabricated and demonstrated in verifying different sign languages of the sentence of “I am”, “happy”, “to meet”, and “you” with triboelectric current signals. Recently, as shown in Figure 6e, Wen et al. proposed a sign language recognition and communication system comprising triboelectric smart gloves, AI block, and the VR interaction interface, and realized the recognition of 50 words and 20 sentences [60]. The triboelectric smart gloves include 15 triboelectric sensors mounted on ten fingers, left and right wrist, left and right palms, the index and middle fingertips of the right hand. Thus, the signal similarity and correlation analysis of the total 50 gestures are carried out based on the original data collected by these fifteen sensors, and the method of non-segmentation enables the system recognition of words or single gestures, and sentences or continuous multiple gestures, with high accuracies of 91.3% and 95%, respectively. Then a method of segmentation, referring to dividing all the sentence signals into word fragments, is developed to recognize new/never-seen sentences and obtains an accuracy of 85.58%. In the demonstration displayed in an embedded VR interface, the user can communicate with non-signer via the triboelectric smart gloves transmitting complete sentences. For depth sensing, Zhang et al. proposed a tailorable, TENG-based artificial perception and transmission nerve (APTN), as shown in Figure 6f [241]. By constructing the gradient thickness in the dielectric layer, different outputs at different positions are applied to locate and transmit simulated mechanosensitive signals to the driver circuit, which realize the main functions of the biological sensory and nervous system. Thus, an APTN-based robotic hand is further demonstrated, and a selected gesture is achieved as sliding designed locations, which shows great potential in real-time control of prosthetic arms for disabled users.

In addition, the biomechanical energy from organs also can be utilized to develop self-powered implantable electronics [245,246]. Ouyang et al. reported a symbiotic cardiac pacemaker (SPM) based on an implantable TENG (iTENG) [247]. This iTENG has a core-shell structure, which consists of two triboelectric layers (nanostructured PTFE thin film and Al film with a spacing of 500 μm), supporting structure (a memory alloy ribbon), and the shell with two encapsulation layers (a flexible Teflon film and a PDMS layer). The entire SPM system including the iTENG, a power management unit (PMU), and a battery-less pacemaker unit is implanted into the chest of a pig (Adult Yorkshire porcine, male, 35 kg), a capacitor is firstly charged from 0 V to 4V within 63 min under the blood pressure of ~110/60 mmHg and heart rate of ~82 bpm, then the SPM system is switched on by a wireless passive trigger. The pacing therapy is observed promptly, heart rate remains at ~68 bpm, blood pressure recovers to the previous level before sinus node hypothermia created with an ice cube. Similarly, Li et al. introduced an implantable piezoelectric energy generator (iPEG) consisting of one elastic skeleton and two piezoelectric composites [248]. In the Vivo test, the iPEG is anchored at the cardiac apex, and a battery-less pacemaker can be powered and generate a continuous pacing ECG pulse with an amplitude of ∼2.5 V and a pulse width of ∼2 ms.

To sum up, monitoring the human body with self-powered sensors has demonstrated the advantages of low cost and sustainability. Additionally, artificial intelligence-enabled sensing has provided humanistic concern to the elderly and disabled users. As well as wearable solutions discussed before, the human body microgrid based on EHs harvesting energies from the body’s movements, temperature, and sweat, can be developed to fit with commercial electronics. Moreover, the development of electric skins fabricated with EHs can be a potential direction for achieving an adaptive self-powered monitoring system. Looking into practical healthcare applications, besides diagnosis from data analysis based on equipped self-powered sensors, self-powered therapies of drug, light, heat, and electrical stimulation need in-depth studies and verifications.

### 4.4. Robotics

As a strong driving force of productivity and efficiency improvement, robots and robotic manipulators have been research hotspots for smart manufacturing and the smart industry. Although most frontier works are focusing on robots’ humanity with electronic skin, there are still development spaces for self-powered sensors functionalizing robotic parts with TENG or PENG [249,250,251]. For example, Zhang et al. fabricated a stretchable sensor array attached to the robotic palm as multifunctional electronic skin, in which the sensor array under capacitance mode is applied for proximity and high-pressure sensing, and the sensor array under TENG mode is applied for location and low-pressure sensing [252]. Moreover, the developments of energy harvesting technology also show new strategies for robotic controlling by energy-autonomous feedback [253,254].

**Figure 7:**
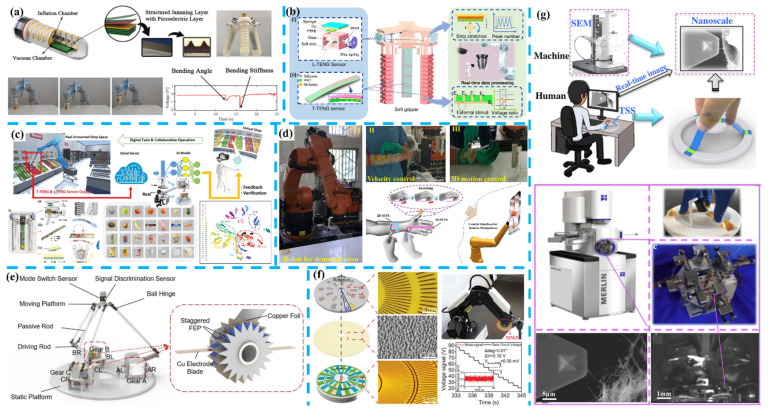
Robotics. (**a**) Flexible self-powered multifunctional sensor for stiff-ness-tunable soft robotic gripper (Reprinted with permission from ref. [255]. Copyright 2021 Elsevier). (**b**) Triboelectric nanogenerator sensors for soft robotics aiming at digital twin applications (Reprinted with permission from ref. [6]. Copyright 2021 Nature). (**c**) Artificial Intelligence of Things (AIoT) enabled virtual shop applications using self-powered sensor enhanced soft robotic manipulator (Reprinted with permission from ref. [259]. Copyright 2021 John Wiley & Sons). (**d**) Triboelectric self-powered wearable flexible patch as 3D motion control interface for robotic manipulator (Reprinted with permission from ref. [260]. Copyright 2018 American Chemical Society). (**e**) A Delta-parallel-inspired human-machine interface by using self-powered triboelectric nanogenerators toward 3D and VR/AR manipulations (Reprinted with permission from ref. [261]. Copyright 2020 American Chemical Society). (**f**) A self-powered angle sensor at nano-radian-resolution for robotic arms (Reprinted with permission from ref. [262]. Copyright 2020 John Wiley & Sons). (**g**) Intuitive-augmented multidimensional nano-manipulation terminal using triboelectric stretchable strip sensors (Reprinted with permission from ref. [263]. Copyright 2019 Elsevier).

In Figure 7a, Xie et al. introduced an embedded multifunctional self-powered sensor consisting of a PVDF film and a soft micro-structured jamming layer, into a fully 3D printed hard-reinforced soft finger with a pneumatic body [255]. The embedded sensor plays as a feedback self-powered sensing element for curvature and stiffness, resulting in a sensitivity of 0.55 and 0.09 V m/N respectively. Additionally, the sensor is also an active jamming element in tuning the finger’s stiffness under different inflation and vacuum pressure and does not influence the finger’s movement. Moreover, Zhu et al. introduced a self-powered triboelectric curvature sensor into a soft finger [256], Chen et al. built a series of triboelectric sensors inside the chamber of a soft actuator [257], and Chen et al. proposed a soft gripper equipped with triboelectric skin [258]. Recently, as shown in Figure 7b, Jin et al. introduced a smart 3D printed soft-robotic gripper system based on 15 TENG sensors capturing the continuous motion and tactile information of three actuator fingers [6]. These TENG sensors are clarified into two types, including the patterned-electrode tactile TENG (T-TENG) sensor sensing sliding, contact position, and gripping mode of the soft gripper, and the length TENG (L-TENG) sensor sensing bending motion. The drawback from variable humidity and temperature has been minimized by the readout strategy of peak counting and output ratio. With the aid of the machine learning technology processing raw data in 15 channels, the smart gripper successfully perceives and recognizes 16 objects such as fruits and snacks with an accuracy rate of 98.1%. Then a digital twin unmanned warehouse system in virtual space is demonstrated, in which the objects gripped by the soft gripper in reality are recognized by the pre-trained support vector machine model and trigger the virtual objects gripped and placed in the corresponding boxes. Moreover, as shown in Figure 7c, Sun et al. proposed an artificial intelligence of things (AIoT) enabled virtual shop applications using self-powered sensors enhanced soft robotic manipulator [259]. The patterned-electrode tactile TENG (T-TENG) sensors and the length TENG sensors are equipped to sense motions of the gripper, and 15 channels’ grasping data are collected and trained in the 1D-CNN ML algorithm for automatic feature extraction, and calculated accuracy for six spherical objects and three oval objects can reach up to 96.1%. In addition, a PVDF sensor attached to the tips of fingers for temperature sensing is also developed by its pyroelectric outputs. Thus, the robotic gripper gives users a more comprehensive understanding of the product and a virtual shop for real-time synchronizing user’s intended item selection in the virtual space and robotic arm’s grasp and recognition in the real space.

The benefit from this smart robotic system with real-time shape/size/temperature perception, cups of coffee, and canned drinks with two different temperatures are recognized and digital. Taken as a prospect, the robotic grippers with multifunctional self-powered sensors are promising to facilitate intelligent industrial automation and online shopping.

In Figure 7d, Chen et al. reported a triboelectric-based self-powered wearable flexible patch as a 3D motion control interface for a robotic manipulator [260]. The trajectory recording and fingertip motion sensing can be achieved by a one-dimensional sensor for out-of-plane robotic movement control and a two-dimensional sensor for in-plane robotic movement control. These two sensors are based on silicone rubber with different starch-based hydrogel PDMS elastomer electrodes. An additional 3D printed grid structure is placed on the two-dimensional sensor to achieve efficient contact-separation, and the trajectory and displacement can be calculated by the ratios of voltages in the opposite electrodes, and the velocity of the motion can be calculated by combining with the voltage interval time. For the one-dimensional sensor, the trend of voltages’ ratios calculated for a certain point under different stretching conditions are utilized to provide out-of-plane controlling commands. In the demonstration, a robotic manipulator applied in a smart factory is successfully controlled to touch the whiteboard and write different letters. Recently, Hou et al. fabricated a self-powered delta-parallel human-machine interface (DT-HMI) for 3D sensing and VR/AR manipulations [261]. The DT-HMI gets three parallel branch chains connecting a moving platform and a base platform, and three TENG sensing gears with two copper–electrode blades for detecting clockwise/anti-clockwise rotational angles. A mode-switch sensor and a trigger discriminating sensor are attached to the operating handle to expand the DT-HMI to 6-DOF control. A series of demonstrations are achieved, such as alphabet writing, wireless submarine control, virtual vehicle control, virtual tour, and AR surgery training program, which have proved the DT-HMI as an efficient controller defining 512 commands through low-cost self-powered sensors.

Besides the above examples for improving feasibility and functionality, energy harvesting technologies also are applied to improve precision. As shown in Figure 7f, Wang et al. developed a highly sensitive triboelectric self-powered angle sensor (SPAS) at nanoradian-resolution for robotic arms [262]. For its construction, two rotary contact-sliding mode TENG devices are coaxially assembled, and a difference in the overlaps of radially arrayed electrodes between TENG A and B is designed in the rotator to generate a set of quadrature signals for sensing. As a result, the SPAS obtains the highest resolution of 2.03 nano-radian with optimized electrodes of 1°. Then three SPASs are integrated into three joints in a palletizing robotic arm to record induced voltage signals during movements following a calligraphic script, and that robotic arm can duplicate the calligraphy based on the recorded data while only a 20 μm difference in the width scale obtained in the result. Furthermore, as shown in Figure 7g, Chen et al. introduced triboelectric stretchable strip sensors (TSS) to build an intuitive-augmented human-machine multidimensional nano-manipulation terminal for scanning electron microscope (SEM) equipment [263]. The TSS is composed of three symmetric sensor strips fixed on the base and a mobile stage and can sense three degrees of freedom of linear motion and two degrees of freedom of rotational motion. Thus, the manipulator in SEM can be controlled with the TSS as a terminal and achieve accurate operations of CNTs in micro-scale. Therefore, either robotics grippers or manipulators have been intellectualized by applying self-powered sensors with optimized designs.

For robotics, self-powered sensors have provided accurate information during operating. As for practical applications in smart factories or shops, good stability and reproducibility need further verified in variational temperature, humidity, or potential disturbance from other machines. Moreover, wireless connection and remote control should be studied with low latency and low drop rate in promoting self-powered wireless nodes to achieve unmanned factories or shops. The feedback system based on self-powered sensors is a promising breakthrough in AR/VR scenarios, which enhance interactivity for users and facilitate adjustability for machines.

### 4.5. Transportation

Transportation in smart cities, like nerves in the human body, not only deliver things but also information. Various energy harvesters integrated into roads, bridges or railways, and equipped on vehicles or trains have been reported by applying TENG [264,265,266,267] and PENG [268,269,270], in which vibrations and impacts are converted into electric power to support electronics monitoring transportation. Along with further signal processing, self-powered sensors developed from energy harvesting technologies also provided crucial information for safety monitoring and early warning.

**Figure 8:**
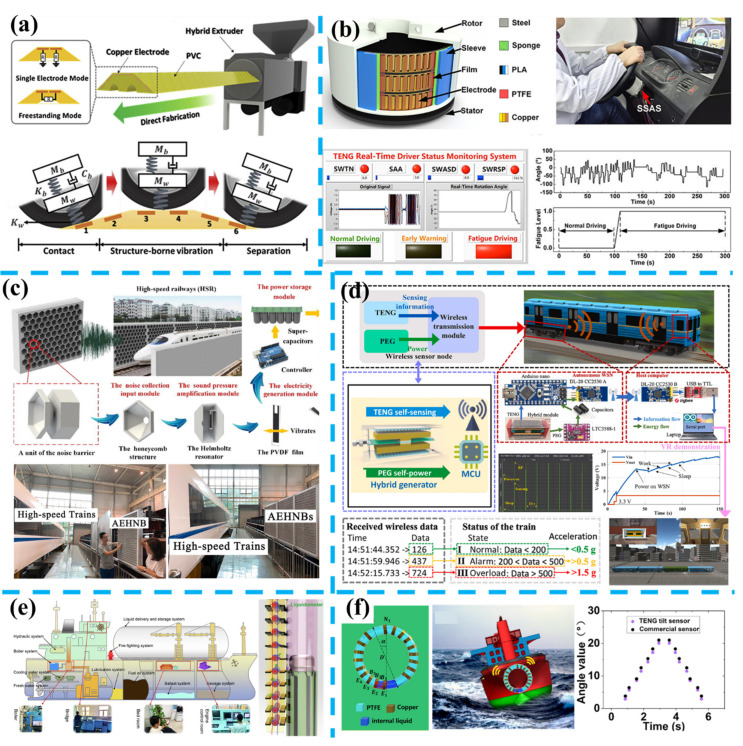
Transportation. (**a**) Triboelectric speed bump as a self-powered automobile warning and velocity sensor (Reprinted with permission from ref. [271]. Copyright 2020 Elsevier). (**b**) TENG enabled a real-time monitoring system of automobile driver status and intelligent fatigue warning (Reprinted with permission from ref. [273]. Copyright 2021 American Chemical Society). (**c**) A renewable low-frequency acoustic energy harvesting noise barrier for high-speed railways (Reprinted with permission from ref. [274]. Copyright 2018 Elsevier). (**d**) Self-sustained autonomous wireless sensing based on a hybridized vibration mechanism (Reprinted with permission from ref. [22]. Copyright 2021 Elsevier). (**e**) Reliable mechatronic indicator for self-powered liquid sensing toward smart manufacture and safe transportation (Reprinted with permission from ref. [275]. Copyright 2020 Elsevier). (**f**) A robust and self-powered tilt sensor for ship attitude sensing (Reprinted with permission from ref. [276]. Copyright 2020 Elsevier).

In Figure 8a, Heo et al. applied triboelectric technology to develop a speed bump as a self-powered automobile warning and velocity detecting sensor [271]. This triboelectric speed bump comprises a commercial PVC speed bump as a body substrate and six copper electrodes inside for sensing. As a vehicle passes over this speed bump, the triboelectric sensor will generate peaks of contact and leave, the time interval between two peaks and peaks’ value can be utilized to estimate the vehicle’s velocity. In addition, negative affect from water and soil contamination on this triboelectric speed bump will be eliminated by multi times friction with the vehicle’s rubber tires. In practice, a self-powered pedestrian warning system is demonstrated, in which 120 LEDs placed near the pedestrian are lighting as the vehicle moves over the triboelectric speed bump. Furthermore, Zhang et al. fabricated concrete beams with embedded TENG rebars as proof prototypes of structural elements [272].

The TENG rebars can detect damage patterns in concrete beams at multiscale and harvest vibration energy, which is promised to be the next generation of scalable, cost-effective, and multifunctional structural elements for smart transportation. Moreover, Tang et al. developed a fully self-powered instantaneous wireless traffic monitoring system based on TENG and magnetic resonance coupling [277]. In this system, a TENG integrated with an L-shaped switch is used to convert the energy from people’s stepping or electric motorbike’s rolling over, a diode, an LC oscillating circuit transmitter module with a capacitive pressure sensor, and a receiving module with a signal processor. This wireless monitoring system can be applied to count the flow rate by counting the received information in a duration, measuring the electric motorbike’s speeds with an accuracy of 94%, and judging the direction of the motorbike on the non-motorized lanes.

Besides roads, Feng et al. proposed a self-powered smart safety belt enabled by two types of TENGs for driving status monitoring. Two sensor arrays composed of arch-shaped TENGs are attached to the shoulder and waist location of the safety belt’s diagonal strip, to monitor the turning directions and angles of the driver. These electric signals are collected to analyze the driving status and prevent drivers and passengers from traffic accidents. Similarly, the self-powered sensors also have been mounted on the accelerator and brake to monitor driving behavior, which has been reported by Meng et al. [278] and Lu et al. [279]. Recently, Xu et al. developed a smart monitoring system for automobile drivers by combing a self-powered steering-wheel angle sensor (SSAS) and a signal processing unit, as shown in Figure 8b [273]. The SSAS is based on a three-phase rotating-typed TENG integrated into the steering wheel and obtains a high resolution of 1°. With data collected from five volunteers driving simulation tests under normal and fatigue driving, the warning thresholds of four parameters, including SWTN (number of steering wheel turns), SAA (averaged steering angle), SWASD (standard deviation of the steering wheel angle), and SWRSP (Proportion of time spent with steering wheel remaining stationary), are calculated for judging fatigue level in a real driving demonstration.

For railways, as shown in Figure 8c, Wang et al. reported a Helmholtz resonator with a PVDF film as a renewable low-frequency acoustic energy harvesting noise barrier [274]. In the prepared prototype, four main components of the noise collection input module, the sound pressure amplification module, the electricity generator module, and the power storage module are mounted together. The output voltage increases with increasing sound pressure, and a voltage of 74.6 mV and a power of 1.24 μW are obtained at 110 dB. Wang et al. introduced a multi-functional wind barrier based on TENG equipped along roads or railways [280]. There are 66 TENG units based on FEP membrane fluttering integrated into the prototype. The flutter frequency of the membrane is proportional to the incoming mean wind speed, which implies that the TENG unit can be a self-powered wind speed sensor. Moreover, Zhang et al. introduced a rotating typed wind energy harvester based on double-layer elastic rotation TENG (ER-TENG) to monitor high-speed moving trains, and the ER-TENG is demonstrated to power commercial traffic lights and hygrothermograph under the simulated wind.

For the trains, Jin et al. introduced a self-powered wireless smart sensor based on a hybridized electromagnetic-triboelectric nanogenerator for train monitoring [281]. The system is powered by vibration-induced electric power and transmits real-time data of temperature and humidity to a cellphone. In Figure 8d, Wang et al. Wang et al. reported a self-sustained autonomous wireless sensing node (WSN) for train monitoring by applying a hybrid TENG and PEG vibration module [22]. A demonstration of train status monitoring in virtual space is operated with a shaker. The PEG part is applied in energy harvesting with an LTC-3588–1 based power management circuit and two energy storage capacitors. The TENG part is applied to collect vibration information by an Arduino Nano development board, then wirelessly transmit it through an RF transceiver and display it on a computer building virtual train. While the autonomous WSN communicates with the computer-based on the Zigbee in IEEE 802.15.4 international standard, bigger received data present a heavier vibration acceleration state of the train.

For the ships, as shown in Figure 8e, An et al. reported a TENG-based block-inserting mechatronic (BMI) indicator panel for liquid level sensing [275]. The flaps of the TENGs can flip from one side to the other side gradually as the magnetic floater inside is rising or falling by the buoyancy of liquid level changing. Then the position of the magnetic floater can be visually recognized and remotely transferred based on the TENGs’ signals. A complete liquid level control system and liquid flow monitoring system have been established for automatic control, wireless monitoring of water level and flow rate in real-time, indicating practicability in smart maritime transportation such as real-time reading water level information of a boiler feed tank in the bridge or engine control room. Wang et al. developed an electromechanical integrated tilt sensor based on liquid–solid interfacing TENG, as shown in Figure 8f [276]. By calculating the voltage signals generated by pure water flowing inside an annular PTFE tube with copper electrodes segment disposed on its surface, the real-time ship tilt angle is obtained by this self-powered tilt sensor and the accuracy agrees well with a commercial sensor.

For future smart transportation, either self-powered sensors integrated into conveyances or roads, durability is an important consideration. The current strategy of fabrication self-powered sensing unit on structural materials has been proved efficiently. The artificial intelligent enabled monitoring based on multiple self-powered sensors is promised to improve security level and automatic pilot. Moreover, EHs located along roads also can help build self-sustained IoT systems to record conveyances’ dynamic information and wirelessly transmit roads’ conditions to users for route planning.

### 4.6. Blue Energy

**Figure 9:**
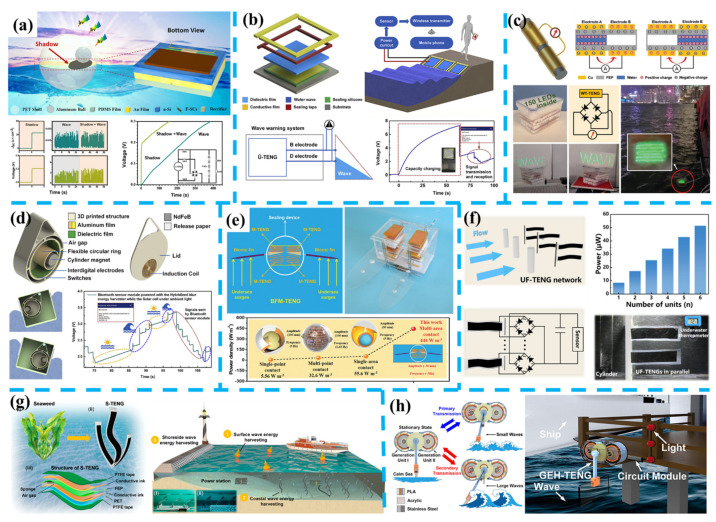
Blue energy. (**a**) Shadow enhanced self-charging power system for wave and solar energy harvesting from the ocean (Reprinted with permission from ref. [284]. Copyright 2021 Nature). (**b**) Thin-film blue energy harvester and seashore IoT applications (Reprinted with permission from ref. [289]. Copyright 2019 Elsevier). (**c**) Multi-mode water-tube-based blue energy harvester (Reprinted with permission from ref. [290]. Copyright 2021 John Wiley & Sons). (**d**) Hybridized blue energy harvester aiming at all-weather IoT applications (Reprinted with permission from ref. [291]. Copyright 2020 Elsevier). (**e**) Bionic-fin-structured TENGs for undersea energy harvesting (Reprinted with permission from ref. [292]. Copyright 2020 John Wiley & Sons). (**f**) An underwater flag-like TENG for harvesting ocean current energy (Reprinted with permission from ref. [293]. Copyright 2019 Elsevier). (**g**) Flexible seaweed-like TENG for marine IoT applications (Reprinted with permission from ref. [294]. Copyright 2021 American Chemical Society). (**h**) Ocean wave graded energy harvesting and condition monitoring with TENG (Reprinted with permission from ref. [295]. Copyright 2021 American Chemical Society).

Coastal cities as economic and cultural centers urgently need energy transition, especially for realizing carbon neutrality. Thus, extracting blue energy from the ocean, which covers 71% of the Earth’s surface and 90% of the Earth’s biosphere, should be one of the most efficient solutions. Accordingly, blue energy harvesters have been developed to facilitate self-sustainable monitor systems along coastlines and in the ocean [282,283]. In Figure 9a, Zhang et al. introduced the shadow effect and hybrid mechanism to enhance a self-charging power system in the ocean [284]. In a transparent ball, a shadow-tribo-effect TENG interacts with a small Al ball rolling with water waves, resulting in a peak power density of 718 μW/cm^2^ by stimulated waves and light operating on this hybrid energy system. Then the fiber-supercapacitors are charged, and results show that this hybrid energy system gets superiority over individual energy harvesting technology. The spherical structural blue energy harvesters attract most research attention for harvesting wave energy from all directions [285,286,287]. For coastline, Cheng et al. reported another atmospheric pressure difference driven TENG for coastline, where water waves first act on a soft membrane then release the energy in form of airflow to trigger a flutter-driven TENG at lower speed airflow and a disc-shaped TENG at stronger airflow [288]. As shown in Figure 9b, Liu et al. reported a thin film blue energy harvester based on external electrodes based TENG [289]. Benefit from the Bar electrode and U-shaped electrode, the shielding effect from the water environment is hugely minimized, and outputs are effectively improved. Besides extracting electric power from waves’ flood and ebb processes, a wave warning system based on Bar electrode playing as a safety switch, a continuous powering system, and a wireless transmission system based on a Bluetooth module, are demonstrated for a self-sustainable IoT-based seashore.

Similar to liquid–solid contact TENG, Wu et al. prepared a water-tube-based TENG for blue energy harvesting in Figure 9c [290]. By encapsulating deionized DI water into an FEP tube and two Cu electrodes wrapped side by side outside the tube, this TENG unit can efficiently generate electric power by shaking, rotating, swinging. With an ultrahigh volumetric output charge density of 9 mC/m^3^ at a frequency of 0.25 Hz, a power package with 34 units is floating in the harbor and successfully lights 150 LEDs. There are also buoy-based blue energy harvesters floating on water to extract electric power [296,297,298,299]. In Figure 9d, Liu et al. proposed a hybridized blue energy harvester with a pendulum design packaged in a sealed box [291]. As the box swings with water waves, inside cylindrical magnet will roll with that pendulum structure, thus generating electric signals by the TENGs and EMG. With related hybridized circuits, outputs from TENGs and EMG have been converted to DC output to charge a lithium battery or power a digital temperature sensor. When integrated with a Bluetooth sensor module and solar cells, this hybridized blue energy harvester facilitates an all-weather IoT platform for wireless data transmission under conditions of with or without daylight and water waves.

Other hybridized blue energy harvesters based on rotation structures [300], swing structures [301,302,303,304], spheroidal structures [305,306], are also developed in electromagnetic-triboelectric hybridized mechanisms to enhance outputs and expand applications.

Besides blue energy harvesters floating in the ocean, Chen et al. designed a bionic-jellyfish TENG with a hermetic and elastic resilience package [307], which can harvest energy based on the liquid pressure-induced contact-separation of the triboelectric layers during raising or lowering in water. The results show that this device can supply power for dozens of green LEDs, a temperature sensor, or a wireless fluctuation sensor in a self-powered early-warning system. Recently, Zhang et al. proposed another undersea device based on bionic-fin-structure assisted with multilayer-structured TENG (BFM-TENG) in Figure 9e [292]. A 3D printed hollow wedge-shaped cone is applied to convert kinetic motions from bionic-fin-structure to the TENGs’ contact-separation processes. As a result, this BFM-TENG has delivered an output power of 444 W/m^3^, which is 1–2 orders of magnitude higher than previously reported spherical devices [308,309,310]. Another design of underwater flag-like TENG (UF-TENG) is reported by Wang et al., as shown in Figure 9f [293]. The UF-TENG is fabricated by sealing one strip of PTFE membrane between two conductive ink-coated PET membranes with waterproof PTFE tape. Thus, triboelectric layers in the UF-TENG will realize contact separation via enhanced vibrations under the Karman vortex street induced by an external cylinder structure. A peak output power of 52.3 μW is achieved with six UF-TENG units at the flow velocity of 0.461 m/s, and a thermometer is continuously powered. As shown in Figure 9g, Wang et al. reported a flexible seaweed-like TENG (S-TENG) capable of harvesting wave energy when integrated into oceanographic buoys, undersea power stations, and coastlines [294]. Recently, as shown in Figure 9h, Xu et al. reported a graded energy harvesting TENG (GEH-TENG) to adapt to changeable waves by two generation units operating in different transmission states [295]. Hence the GEH-TENG help monitor ocean wave condition and light caution lights installed on the shore. To sum up, energy harvesters equipped on coastlines, submerged in water, and floated in oceans, can help build power stations and self-powered/self-sustainable IoT applications.

Blue energy has become an important direction for renewable energy and promises scaled power stations for self-sustained IoT platforms in oceans. In regard to the costs, waterproofing and anticorrosive factors should be taken into account. In addition, mechanical structure and power management also need to be optimized to match irregular water waves and seasonal variations caused by ocean environment changes. The undersea prototypes have provided opportunities for harvesting ocean current energy, but also raised challenges of maintenance and power delivery.

### 4.7. Aircraft and Aerospace

**Figure 10:**
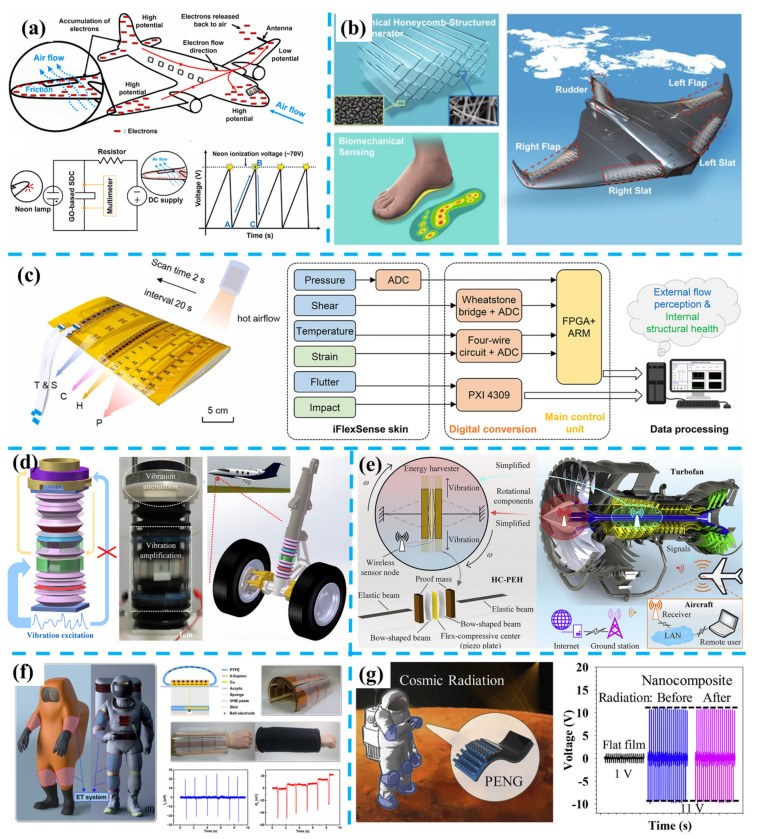
Aircraft and aerospace. (**a**) Aviation static electricity harvesting and storage for the aircraft (Reprinted with permission from ref. [312]. Copyright 2020 Elsevier). (**b**) Morphing wing energy harvesting (Reprinted with permission from ref. [313]. Copyright 2021 Springer). (**c**) Intelligent flexible sensing (iFlexSense) skin for multifunctional flying perception (Reprinted with permission from ref. [314]. Copyright 2021 Elsevier). (**d**) Vibrational energy harvesting enhanced by energy transfer and redistribution in the landing gear of a plane (Reprinted with permission from ref. [315]. Copyright 2020 Elsevier). (**e**) Energy harvesting for jet engine monitoring (Reprinted with permission from ref. [316]. Copyright 2020 Elsevier). (**f**) Self-powered electro-tactile system for spacesuit (Reprinted with permission from ref. [317]. Copyright 2021 Science). (**g**) High-performance piezoelectric nanogenerator for energy harvesting and radiation protection in space (Reprinted with permission from ref. [318]. Copyright 2019 Elsevier).

As energy harvesting technology moves into harsh environments such as aircraft and aerospace, reliable materials and structures are crucial. Qing et al. have reviewed the progress of piezoelectric-based structural health monitoring for aircraft, in which piezoelectric sensors mounted or embedded in the aircraft can convert dynamic structure state of damage, load or temperature, into the corresponding electric signals diverse from the baseline [311]. In addition, Fu et al. adopted an event-triggered mechanism based on filtered piezoelectric signals to achieve a low-power high-response wireless structural health monitoring system for impact detection of composite airframes, and this system will enter the sleep mode to save energy when no impact is detected by the piezoelectric sensors. To achieve active energy harvesting, as shown in Figure 10a, Chan et al. introduced graphene oxide thin film structural dielectric capacitors for aviation static electricity harvesting and storage [312]. The prototype can harvest static electricity generated in airflows via accumulated electrostatic charges flowing from a relatively high potential to a site of lower potential and by releasing to air through the antenna. The result proves that harvested energy can power a navigation light, which shows the potential of powering other electronics on the aircraft. In Figure 10b, Tao et al. fabricated a hierarchical honeycomb structured TENG (h-TENG) for aircraft morphing wing energy harvesting [313]. The unique honeycomb-inspired architecture is obtained by thermoplastic molding and wafer-level bonding with multilayered wavy PET/AgNWs/FEP thin films, then treated by a corona discharging process under a ~4 kV voltage for 10 min. As the h-TENG is integrated into the morphing wing of an unmanned aerial vehicle, dynamic signals generated at different excitation frequencies and the wing’s deflected angle can be further utilized as crucial control factors for calibration. Moreover, dozens of LEDs can be continuously lightened up by the h-TENG based morphing wing. Recently, Xiong et al. reported intelligent flexible sensing (iFlexSense) skin for multifunctional flying perception in aircraft, as shown in Figure 10c [314]. The skin fabricated on flexible polyimide substrate obtains sensing area and digital conversion area. The sensing area contains all 71 sensors including 30 piezoelectric sensors (P) for coupled airflow-structural dynamics and impacts locating, 12 hot-film shear stress sensors (H), 21 capacitive pressure sensors (C), 4 resistive temperature sensors (T), and 4 resistive strain sensors. The performances of the skin equipped on a standard airfoil model have been verified in wind tunnel experiments and impact positioning tests, which match with commercial sensors under different velocities and angles of attack. In addition, machine learning has been applied for impacts locating via collected piezoelectric generated data. In brief, the iFlexSense skin has been applied as an easily installed monitoring system for aircraft without destructions.

As shown in Figure 10d, Du et al. reported a two-degree-of-freedom (2DOF) resonant system promised to harvest vibrational energy from the landing gear of a plane [315]. In this resonant system, there is an energy absorber connected to upper and lower springs, and a protected object placed on the top. The springs are fabricated by two silicone rubber-based bellows-tube-shaped TENGs, wherein the nitrile rubber as another triboelectric layer is bonded on one curved surface. Once the absorber absorbs external vibrational energy, it will resonate and drive the springs reciprocating large-amplitude extension–contraction and the TENGs generating electric power. At the excitation of 11 Hz, near to the resonant frequency, the lower TENG delivers a power density of 15.72 mW/m^2^, and the upper TENG delivers a power density of 2.55 mW/m^2^, then an electronic clock is powered by a charged capacitor with two TENGs parallel connected. By calculation, this resonant system equipping with energy harvesting units achieves an amplitude amplification factor of 375% and a 3477% power enhancement, while the amplitude of the protected object decreases by 55%, which shows the potential of functionalizing mechanical structures in the aircraft. In Figure 10e, Wang et al. introduced an energy harvesting unit into jet engine monitoring [316]. The high-efficiency compressive-mode piezoelectric energy harvester (HC-PEH) is fabricated by bonding a soft PZT-5H plate, two bow-shaped beams, two proof mass, and two elastic beams together, and the HC-PEH with a wireless sensor are mounted in the fan nozzle of the Jet engine. When the rotation speed of the jet engine increases to 1000 r/min, generated electric power is sufficient for the transmitter sending a 3-axis accelerometer’s data every 3 s to the computer placed three meters from the Jet engine. Thus, this prototype has proved to support a self-sustainable Jet engine monitoring system and demonstrates practical application value.

In aerospace, as shown in Figure 10f, Shi et al. reported a self-powered electro-tactile (ET) system for the massive space suit [317]. The ET system is based on electrostatic discharging triggered by a series of contact-separation TENGs below the spacesuit. In the demonstration, the prototype is attached to the forearm to mimic the situation of wearing a spacesuit and the ball-shaped electrodes are close to human skin. As a result, the touching or the impact motion on the TENG closely to the suit will drive the ball-shaped electrodes to release charges on human skin to enhance tactile sensation. For energy harvesting in aerospace, Ye et al. reported a high-performance PENG based on micro-structured P(VDF-TrFE)/BNNTs composite in Figure 10g [318]. The prepared piezoelectric device obtains a power density of 11.3 μW/cm^2^ and a sensitivity of 55 V/MPa, and the device successfully lights an LCD screen and power a digital watch. The neutron shielding capability has been investigated using neutron activation analysis, and test results show that the macroscopic absorption cross-section is enhanced by 260% compared to pristine P(VDF-TrFE), and output performance of the PENG remains nearly unchanged after radiation, which proves that the device can be the ideal candidate for scavenging energy under the harsh space environment.

To sum up, applying energy harvesting technologies has enabled sensors self-powered or self-sustained in extreme environments. These developed sensors help monitor aircraft’s flying status and reported prototypes also are proved to harvest energy from airflow and impacts. Along with the increased demand for unmanned aerial vehicles (UAVs) for aerial photo and surveying, self-powered sensors located on airfoils help dynamic changes digitalized for further analysis, and harvested energy enables low-power wireless transmission as well as realize energy-saving for longer operating time. As for aerospace, solar energy and thermal energy can be utilized for the satellite’s sensing, and mechanical energy induced by astronauts and machines can be utilized for the space station’s sensing.

## 5. Conclusions

With the rapid development of energy harvesting technology as well as sensor technology, various sensors with self-powered designs and IoT applications with self-sustained features are promoted to support flourishing smart cities. New applications related to smart homes, human monitoring, robotics, transportation, blue energy, aircraft, and aerospace are illustrated and discussed from mechanism to performance. Besides extracting electric power and information from the above scenarios, the prototypes also show the potentials of collaboration with traditional hardware such as power management integrated circuits, microprogrammed control units, and wireless transmitters. The dynamic environment is one of the most important challenges for developing self-powered or self-sustained applications. On one hand, excitations of mechanical, solar, or thermal, are not sustained and stable in most scenarios, resulting in unstable outputs of corresponding energy harvesters. On the other hand, unexpected contamination or destruction, and exceeded stretching or compression, have aggravated the instability in energy harvesting and sensing. To address these issues, self-cleaning and self-healing materials, reliable mechanical structures, and packages are needed to achieve stable performance and accurate sensing relationships.

Following emerging trends might contribute to the future applications are envisioned: (1) for smart homes, walls and roofs integrated with energy harvesters extracting electric power from rains, winds, solar, and even vibrations, can be a powerful station for systematic controlling and monitoring; (2) human body sensor networks designed with light-weight and trained with artificial intelligent algorithms can be utilized to build a self-sustainable monitoring system for motions and health condition; (3) greenhouses functionalized by large-scaled energy harvesters supporting sensors and controllers can real-time monitor plants growth and wirelessly control related conditions of irrigation and lighting; (4) early warning of fire or landslide is important, and correspond sensors are placed at forest or mountain area where wind and rain energy can be applied to extend sensors’ service life; (5) to explore the universe, energy harvesters equipped in robotics or astronauts can be useful sensing units and supplementary power units for continuously marching and exploration.

With the growing and in-depth studies of energy harvesting technologies and sensor technologies, there will be more profound discoveries and progresses for constructing a smarter city of self-sufficient energy and intelligence operation in near future.

## Data Availability

Not applicable.
